# Thymidine phosphorylase induction by ionizing radiation antagonizes 5-fluorouracil resistance in human ductal pancreatic adenocarcinoma

**DOI:** 10.1007/s00411-022-00962-w

**Published:** 2022-01-27

**Authors:** Lucas D. Lee, Ioannis Pozios, Verena Liu, Silke B. Nachbichler, Dirk Böhmer, Carsten Kamphues, Katharina Beyer, Christiane J. Bruns, Martin E. Kreis, Hendrik Seeliger

**Affiliations:** 1 Department of General and Visceral Surgery, Charité—Universitätsmedizin Berlin, Corporate Member of Freie Universität Berlin and Humboldt-Universität zu Berlin, 12200 Berlin, Germany; 2 Department of Radiotherapy and Radiation Oncology, Klinikum der Universität München, 81377 Munich, Germany; 3 Department of Radiation Oncology and Radiotherapy, Charité—Universitätsmedizin Berlin, Corporate Member of Freie Universität Berlin and Humboldt-Universität zu Berlin, 12200 Berlin, Germany; 4 Department of Surgery, University Hospital of Cologne, 50937 Cologne, Germany; 5 IU Health University, 10243 Berlin, Germany

**Keywords:** Pancreatic cancer, 5-fluorouracil, Thymidine phosphorylase, Irradiation, Chemotherapy resistance

## Abstract

Chemoresistance in pancreatic ductal adenocarcinoma (PDAC) frequently contributes to failure of systemic therapy. While the radiosensitizing properties of 5-fluorouracil (FU) are well known, it is unknown whether ionizing radiation (IR) sensitizes towards FU cytotoxicity. Here, we hypothesize that upregulation of thymidine phosphorylase (TP) by IR reverses FU chemoresistance in PDAC cells. The FU resistant variant of the human PDAC cell line AsPC-1 (FU-R) was used to determine the sensitizing effects of IR. Proliferation rates of FU sensitive parental (FU-S) and FU-R cells were determined by WST-1 assays after low (0.05 Gy) and intermediate dose (2.0 Gy) IR followed by FU treatment. TP protein expression in PDAC cells before and after IR was assessed by Western blot. To analyze the specificity of the FU sensitizing effect, TP was ablated by siRNA. FU-R cells showed a 2.7-fold increase of the half maximal inhibitory concentration, compared to FU-S parental cells. Further, FU-R cells showed a concomitant IR resistance towards both doses applied. When challenging both cell lines with FU after IR, FU-R cells had lower proliferation rates than FU-S cells, suggesting a reversal of chemoresistance by IR. This FU sensitizing effect was abolished when TP was blocked by anti-TP siRNA before IR. An increase of TP protein expression was seen after both IR doses. Our results suggest a TP dependent reversal of FU-chemoresistance in PDAC cells that is triggered by IR. Thus, induction of TP expression by low dose IR may be a therapeutic approach to potentially overcome FU chemoresistance in PDAC.

## Introduction

Pancreatic ductal adenocarcinoma (PDAC) is the fourth leading cause of cancer-related deaths in western countries (Siegel et al. 2020). Up to 20 percent of PDAC patients present with resectable disease, yet their prognosis remains poor (van Roessel et al. 2020). Overall survival is widely known to be worse in non-curative PDAC patients despite treatment with modern chemo- and immunotherapeutic agents (Conroy et al. 2018). Resistance towards cytotoxic agents, ionizing radiation (IR), or both, confers biological aggressiveness and accounts for poor therapeutic response.

5-fluorouracil (FU) is the backbone of systemic therapy of PDAC in palliative and adjuvant settings (Conroy et al. 2011; Conroy et al. 2018). As a pyrimidine analog, FU exerts its anticancer effects by incorporating its metabolites into DNA and inhibition of thymidylate synthase (TS), a key enzyme of DNA biosynthesis (Longley et al. 2003). Innate or acquired resistance of cancer cells diminishes the effectiveness of most chemotherapeutic agents. To overcome therapeutic resistance towards FU, the mechanisms of FU activation and development of resistance need to be addressed.

Thymidine phosphorylase (TP) is a key enzyme of the FU metabolism and plays a dual role in cancer development and therapy. On the one hand, TP promotes tumor growth and progression by preventing apoptosis and inducing angiogenesis via converting thymidine into its metabolite 2-deoxy-d-ribose (dRib), which has angiogenic properties (Bronckaers et al. 2009; Dikici et al. 2019; Seeliger et al. 2004). On the other hand, TP is necessary for the conversion of FU into its biologically active metabolite 5-deoxyuridine monophosphate (FdUMP) (Schuller et al. 2000), so TP induction combined with these chemotherapeutic agents is beneficial (Bronckaers et al. 2009; Toi et al. 2004). TP is highly expressed in solid tumors including PDAC (Lindskog et al. 2014) and is upregulated by chemotherapeutic agents as well as by IR (Hasegawa et al. 2012).

Low dose IR alters the expression of multiple genes and protects non-tumor cells from injury by subsequent higher irradiation doses through the activation of cell protective signaling pathways (Hou et al. 2015). At the same time, low dose IR has been shown to affect tumor cell proliferation in PDAC and other solid tumors (Liu et al. 2019; Schwarz et al. 2008). Here, we hypothesize that low dose IR induces an upregulation of TP that may antagonize the therapeutic resistance of PDAC cells towards FU.

## Materials and methods

### Cell culture and reagents

The FU-sensitive (FU-S) human PDAC cell line AsPC-1 (ATCC, Rockwell, MD, USA) was maintained in culture as adherent monolayer in Dulbecco’s minimal essential medium (Invitrogen, Karlsruhe, Germany), supplemented with 10% fetal bovine serum (Biochrom, Berlin, Germany) and 1% penicillin/streptomycin (Invitrogen) in 5% CO_2_ in an humidified atmosphere at 37 °C.

### Generation of FU-R variant AsPC-1

An FU resistant (FU-R) variant AsPC-1 was generated by long term in vitro FU exposure as reported before (Ischenko et al. 2008). Stimulation by interferon gamma (IFNγ, Sigma-Aldrich, Taufkirchen, Germany) served as a positive control for TP expression.

### Irradiation of PDAC cells

PDAC cells were irradiated with a Müller RT 250 X-ray device. Plates received either low dose IR (0.05 Gy) at a dose rate of 0.03 Gy/min (225 kV, 5 mA) or intermediate dose IR (2.0 Gy) at a dose rate of 1 Gy/min (225 kV, 15 mA). A 0.35 mm copper-filter was used to absorb lower energy X-rays.

### Proliferation assay

Cells were seeded in 96 well plates (1 × 10^4^ cells per well) 24 h prior to treatments. To assess cell proliferation, cells were assessed after 48 h using a WST-1 assay kit (Roche, Mannheim, Germany) as per manufacturer’s instructions. Absorbance was measured at a wavelength of 450 nm on a microplate reader (Bio-Rad Laboratories, Hercule, CA, USA). IC_50_ was calculated according to standard guidelines.

### Thymidine phosphorylase gene silencing

Total RNA isolated from AsPC-1 cells that were treated with interferon-γ using RNeasy mini kit (Qiagen, Hilden Germany) and subjected to single step RT-PCR using SuperScript III One-Step RT-PCR System with Platinum Taq High Fidelity (Thermo Fisher, Dreieich, Germany). The primer sequences for TP si forward (TCGTGGCCGCTGTGGTGAATGG) and TP si reverse (GCTCCCGGGCCTGCTCCTGGTT) were designed to amplify a 582 base pair PCR product specific for TP. siRNAs were generated using BLOCK-iT RNAi TOPO Transcription Kits and BLOCK-iT Dicer RNAi Kits (both Thermo Fisher) according to the manufacturer’s instructions. Lipofectamine 2000 (Thermo Fisher) was used for transfecting AsPC-1 cells with the designed siRNAs. Control cells were transfected with siRNAs specific for the lacZ reporter gene which was generated in the same way using primers and templates supplied with the kit. Efficiency of TP silencing was confirmed by Western blotting.

### Western blotting

Cells were washed with ice-cold phosphate-buffered saline and resuspended in RIPA buffer supplemented with protease/phosphatase inhibitors (Roche, Mannheim, Germany) to a final concentration of about 10^7^–10^8^ cells per milliliter. Equal amounts of protein were run on polyacrylamide gels, transferred to polyvinylidene difluoride membranes (Millipore, Billerica, MA, USA) and detected using an enhanced chemiluminescence system (Amersham, Braunschweig, Germany). Antibodies for TP (Cell Signaling, Frankfurt, Germany) and β-actin (Sigma-Aldrich) were used according to the manufacturers’ instructions. Blots were processed with ECL plus Western blotting detection kit (Amersham) and the signal was detected using an LAS-3000 image analyzer (Fuji, Tokyo, Japan). Densitometry was performed using an AlphaImager (Alpha Innotech, San Leandro, CA, USA).

### Statistical analysis

All experiments were performed three times independently. The data obtained were expressed as mean ± standard deviation. Statistical evaluation was performed using the paired Student’s *t* test with *p* < 0.05 considered to be significant.

## Results

### FU resistance of PDAC cells

**Fig. 1:**
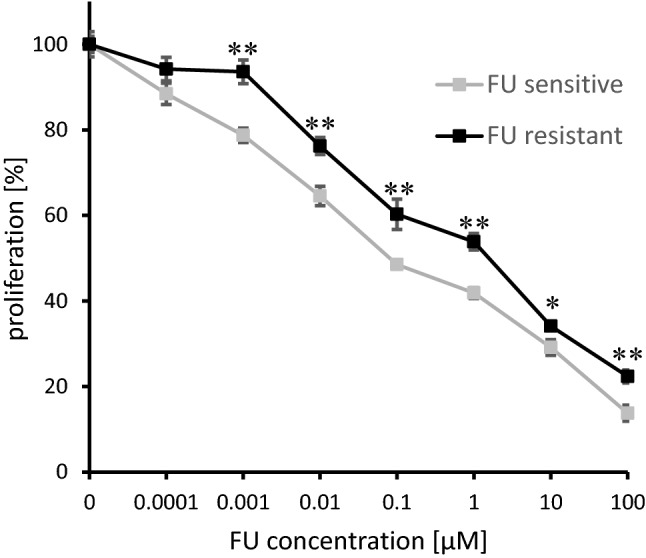
Proliferation of FU sensitive (grey) and FU resistant (black) AsPC1 human PDAC cells in response to FU application. FU inhibited cell proliferation more effectively in FU sensitive than in FU resistant cells over a dose range of 0.001–100 µM (*p < 0.05, **p < 0.01). Experiments were performed three times independently and data points are mean ± standard deviation

To quantify the acquired chemoresistance of PDAC cells following prolonged FU exposition, cells were treated with increasing doses of FU, and proliferation was determined. Chemoresistance was apparent at all doses applied. FU-R PDAC cells showed a 2.7-fold increase of the IC_50_ after FU exposure compared to native PDAC cells (0.471 µM versus 0.172 µM, Fig. 1).

### IR inhibits proliferation of PDAC cells

**Fig. 2:**
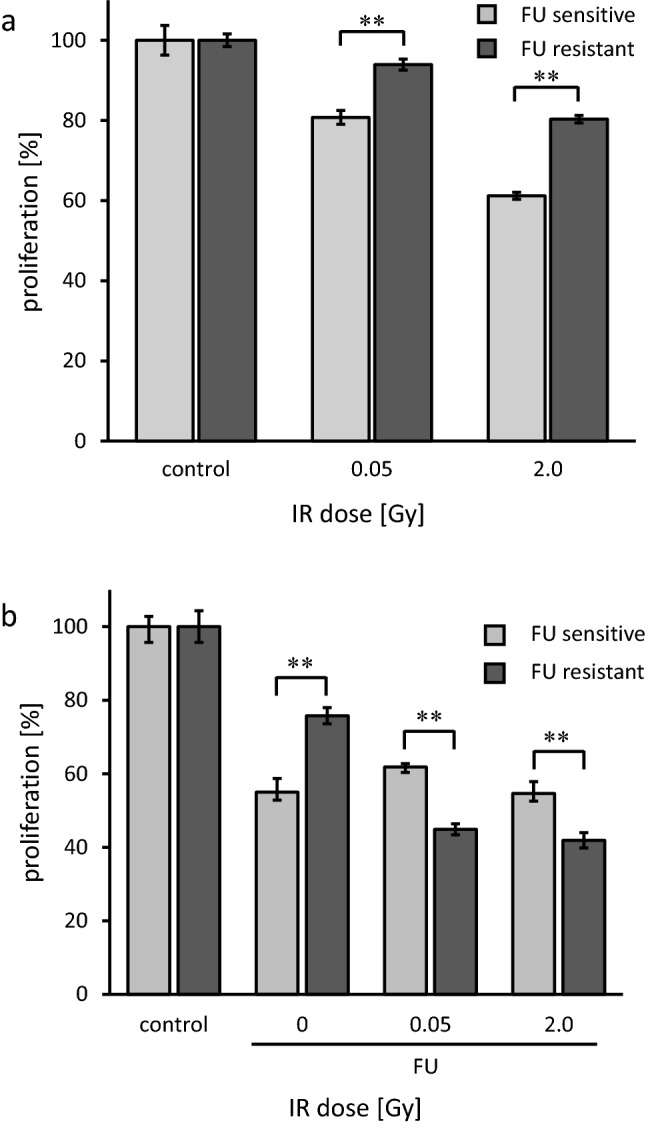
Proliferation of FU sensitive (light grey bars) and FU resistant (dark grey bars) AsPC1 human PDAC cells. **a** Proliferation in response to low dose (0.05 Gy) and intermediate dose (2.0 Gy) IR. FU resistant cells showed concomitant IR resistance towards both doses (**p < 0.01). **b** Proliferation in response to FU was less inhibited in FU resistant cells not previously exposed to IR (**p < 0.01). Following low dose (0.05 Gy) or intermediate dose (2 Gy) IR, FU inhibited proliferation more effectively in FU resistant cells than in FU sensitive cells (**p < 0.01). Experiments were performed three times independently and data points are mean ± standard deviation

IR inhibited the proliferation of both FU-S and FU-R PDAC cells in a dose dependent manner (*p* < 0.01 for 0.05 Gy and 2.0 Gy versus controls, respectively). Compared to parental cells, FU-R cells were less sensitive to IR at both doses tested. Following IR with 0.05 Gy, proliferation was 80.8% of controls for FU-S cells and 93.9% of control for FU-Rcells (*p* < 0.01). Following IR with 2.0 Gy, proliferation was 61.2% of control for FU-S cells and 80.3% of control for FU-R cells (*p* < 0.05, Fig. 2a).

### IR resensitizes chemoresistant PDAC cells towards FU

Without IR, FU reduced FU sensitive cell proliferation to 55.0%, and FU resistant cell proliferation to 75.8% (*p* < 0.01). After exposing cells with 0.05 Gy and 2.0 Gy, respectively, we found an increased FU response in resistant cells. After IR with 0.05 Gy, proliferation was reduced to 61.8% of control for FU-S cells and 44.9% of control for FU-R cells (*p* < 0.01). After IR with 2.0 Gy, proliferation was reduced to 54.7% of control for FU-S cells and 41.9% of control for FU-R cells (*p* < 0.01, Fig. 2b).

### IR upregulates TP protein expression in PDAC cells

**Fig. 3:**
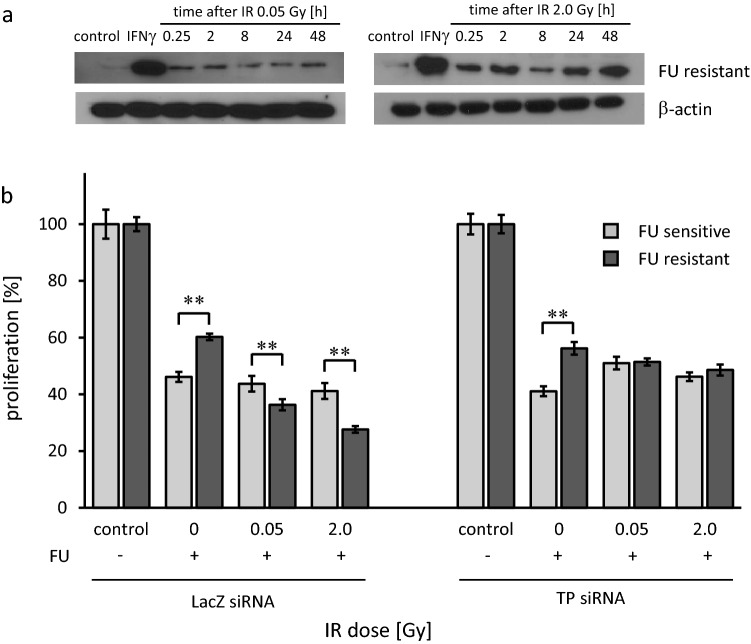
IR upregulates TP and PDAC cell resensitization is dependent on TP. **a** TP protein expression in FU resistant AsPC1 cells following low dose (0.05 Gy) and intermediate dose (2.0 Gy) IR. Both low dose and intermediate dose IR upregulated TP expression in FU resistant cells. **b** Proliferation of FU sensitive (light grey bars) and FU resistant (dark grey bars) AsPC1 human PDAC cells. Anti TP siRNA was used to block TP upregulation, and LacZ siRNA served as control. In control cells, following IR at 0.05 Gy as well as 2.0 Gy, proliferation was inhibited more effectively in FU resistant cells than in FU sensitive cells (**p < 0.01) when challenged with FU. When TP expression was blocked, this effect was abolished. Experiments were performed three times independently and data points are mean ± standard deviation

Western blotting was used after exposing FU-R PDAC cells to IR. IFNγ was used as positive control. Following IR with 0.05 Gy and 2.0 Gy, respectively, a time dependent upregulation of TP protein expression was seen (Fig. 3a).

### PDAC cell resensitization by IR is dependent on TP

To determine the specificity of the resensitization effect, we used siRNA knockdown of TP. After TP silencing, FU-R cells did not show a decrease in proliferation after FU exposure preceded by IR with 0.05 Gy and 2.0 Gy. Proliferation of FU-R cells after FU exposure was 56.2% of control without IR, 59.6% of control for 0.05 Gy and 48.6% of control for 2.0 Gy IR. In contrast, proliferation of FU-R cells was affected by IR in addition to FU exposure in the control group where proliferation of FU-R cells after FU exposure was 60.2% of control without IR (*p* < 0.01), 36.3% of control for 0.05 Gy (*p* < 0.01), and 27.6% of control for 2.0 Gy (*p* < 0.01). In both groups, FU-S cells did show a decrease in proliferation after FU exposure with and without additional IR which was statistically significant (*p* < 0.01, Fig. 3b).

## Discussion

Chemoresistance is a major obstacle to systemic therapy of PDAC with overall response rates to modern FU based regimens found to be about 35% (Adamska et al. 2018; Tong et al. 2018). In this study, we used an FU-S human PDAC cell line and its FU-R variant. Cells of both lines were irradiated with 0.05 and 2.0 Gy prior to FU exposition. Following IR, we noted an increase in 5-FU sensitivity of the FU-R PDAC cells. We could further show that IR upregulates TP which is a key enzyme of the FU metabolism necessary for its biological activation. When TP expression was blocked by siRNA, the resensitization effect of IR on FU-R PDAC cells was mitigated. We thus assumed that this effect of IR on PDAC cells was specifically mediated by TP.

Different tumor-related chemotherapy resistance mechanisms lead to decreased efficacy of cytotoxic drugs. As FU targets intracellular enzymes, its efficiency depends on transport systems including human equilibrative nucleoside transporters (hENTs) and concentrative nucleoside transporters (hCNTs). High expression of hENT1 mRNA leads to FU resistance in PDAC, and inhibition of hENT1 can increase the intracellular FU concentration in human PDAC cells and so enhance cytotoxicity (Wang et al. 2014). Other transport proteins involved in FU uptake include human organic anion transporter 2 (hOat2, SLC22A7). Its expression correlates with chemoresistance in PDAC cells, whereas expression of multidrug-resistance protein 5 (MRP5, ABCC5) is associated with 5-FU sensitivity (Wang et al. 2014). Furthermore, FU resistance is conferred by altered expression of genes involved in cell cycle regulation, proliferation, repair and apoptosis, as DNA and RNA damage caused by FU leads to the activation of DNA repair systems or apoptosis. Several cell survival pathways are involved in FU resistance, including the EGFR/MAPK/ERK, Akt/mTOR, Jak/STAT3, PI3K/NFκB and WNT/GSK3b/β-catenin signaling cascades (Wang et al. 2014). Furthermore, cancer stem cell features and distinct micro RNA expression patterns contribute to chemotherapy resistance in PDAC (Niess et al. 2015; Zhao et al. 2015).

**Fig. 4:**
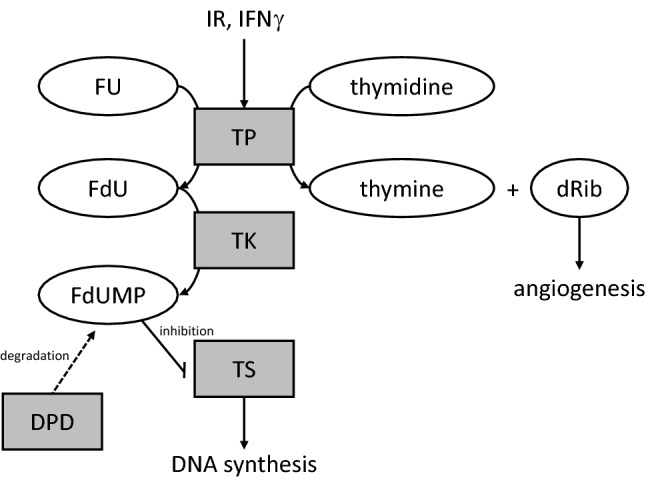
Metabolism of 5-fluorouracil and thymidine phosphorylase

Apart from the more universal resistance mechanisms, specific effects related to intracellular FU metabolism have been found. Two pathways have been identified to synthesize FdUMP from FU: (1) orotate phosphoribosyltransferase converts FU to 5-fluorouridine monophosphate (FUMP) which is converted to FdUMP in several further steps, and (2) TP converts FU to 5-fluoro-deoxyuridine (FdU), which is converted to fdUMP by thymidine kinase (TK). FdUMP itself inhibits thymidine synthase (TS) which leads to the disruption of DNA de novo synthesis. FdUMP then is inactivated by dihydropyrimidine dehydrogenase (DPD) (Wei et al. 1996). Thus, FU resistance can result from an imbalance of FdUMP synthesis and its degradation as well as an overexpression of TS (Fig. 4).

In our experiments, we used a FU-R variant of AsPC-1 PDAC cells that was generated and characterized earlier by our group (Ischenko et al. 2008). Our earlier experiments showed that FU resistance in this system depends at least in part on upregulation of TS mRNA and protein expression that was abolished indirectly by *src* inhibition (Ischenko et al. 2008). Interestingly, there was no difference in DPD expression between FU-S and FU-R cells, suggesting that FU degradation by DPD is not a relevant resistance mechanism at least in the cell lines examined (Ischenko et al. 2008). Based on these data, we then asked whether a modification of TP expression by IR would also alter FU resistance in PDAC cells.

Chemotherapy resistance and IR resistance share phenotypic characteristics, such as the activation of the Jak/STAT3 signaling cascade, loss of Smad4 expression and the expression of multidrug-resistance proteins (Chen et al. 2014; Hou et al. 2014; Oike and Ohno 2020; Wang et al. 2018). In this context, it is not surprising that in our experiments FU-R PDAC cells were radioresistant as well. In line with our findings, clinical and translational studies have proven simultaneous IR and chemotherapy resistance in PDAC and other solid tumors (Orth et al. 2019). FU itself is well established in clinical regimens of combined radiochemotherapy, exploiting its property as a radiosensitizing agent (Buckley et al. 2020).

In contrast, the reverse concept of IR acting as a chemosensitizer is less well defined. Our experiments showed that FU treatment of PDAC cells following different doses of IR results in a significant reduction of tumor cell proliferation in chemoresistant cells. It is remarkable that following IR, FU-R tumor cells responded even better to chemotherapy than the parental FU-S cells. This in turn may result in a higher degree of FU conversion into FdUMP, causing more pronounced cytotoxic effects (Lindskog et al. 2014; Longley et al. 2003). When TP expression was blocked by siRNA, the sensitivity of FU-resistant cells towards IR was eliminated. In line with our results, IR has been shown before to upregulate TP, but the underlying mechanism is not fully understood yet (Derwinger et al. 2013).

Besides examining an intermediate IR dose of 2 Gy, we were interested in the effects of a low dose of 0.05 Gy on FU resistance reversal. Low dose or radioadaptive dosing has been shown to have protective effects on tumor surrounding tissue when afterwards challenged with higher IR doses and increases radiosensitivity of tumor cells (Hou et al. 2015; Schwarz et al. 2008). Since proliferative responses of PDAC cells towards FU were similar after IR at both doses examined, direct IR effects may be less important in our setting than chemosensitizing effects by IR. In fact, radioadaptive IR dosing alters protein expression in non-tumor as well as in tumor cells (Coleman et al. 2005; Hou et al. 2015). Furthermore, IR has been shown to induce inflammation and matrix remodeling, thereby also increasing diverse biomarkers and cytokines (Di Maggio et al. 2015). Thus, it is plausible that IR induces an increase in TP as part of the inflammatory process (Derwinger et al. 2013). Conversely, it has been demonstrated that low dose IR exerts anti-inflammatory responses (Deloch et al. 2018; Schroder et al. 2018). Since inflammatory signaling cascades crosstalk with tumor promoting signaling pathways in PDAC (Pozios et al. 2020; Zhang et al. 2013), attenuating inflammation by low dose IR may have beneficial effects in chemoresistant cells.

Since we did our experiments in vitro in this pilot study, we cannot rule out angiogenic effects of TP confounding the restoration of chemosensitivity in PDAC by IR. However, earlier data of our group indicate that dRib generated from thymidine breakdown by TP exerts a strong angiogenic effect on endothelial cells (Seeliger et al. 2004). Thus, it remains to be established in which way IR interacts with angiogenesis in vivo. Further, effects of the expression of TP in stromal cells in the tumor interstitium remain to be established, as the microenvironment of the tumor can also be involved in the emergence of chemoresistance (Wang et al. 2014).

In conclusion, we were able to show that IR can revert chemoresistance towards FU in PDAC by a specific TP-mediated mechanism. Further experiments should address effects of IR towards the tumor microenvironment and tumor angiogenesis. In perspective, TP directed therapeutic regimens including low dose IR may be beneficial in the clinical treatment of PDAC.

## Data Availability

Raw data and materials are available on request.

## Abbreviations

DPD: Dihydropyrimidine dehydrogenase
dRib: 2-deoxy-d-ribose
FdU: 5-fluoro-deoxyuridine
FdUMP: 5-deoxyuridine monophosphate
FU: 5-fluorouracil
IFNγ: Interferon gamma
IR: Ionizing radiation
PDAC: Pancreatic ductal adenocarcinoma
TK: Thymidine kinase
TP: Thymidine phosphorylase
TS: Thymidylate synthetase
